# Effects of the amount and type of carbohydrates used in type 2 diabetes diets in animal models: A systematic review

**DOI:** 10.1371/journal.pone.0233364

**Published:** 2020-06-12

**Authors:** Anaísa Martins Marques, Bárbara Silva Linhares, Rômulo Dias Novaes, Mariella Bontempo Freitas, Mariáurea Matias Sarandy, Reggiani Vilela Gonçalves

**Affiliations:** 1 Department of Animal Biology, Federal University of Viçosa, Viçosa, Minas Gerais, Brazil; 2 Department of Structural Biology, Federal University of Alfenas, Alfenas, Minas Gerais, Brazil; 3 Department of General Biology, Federal University of Viçosa, Viçosa, Minas Gerais, Brazil; University of Melbourne, AUSTRALIA

## Abstract

Type 2 diabetes mellitus (T2DM) is among the most prevalent diseases in the world, affecting over 420 million people. The disease is marked by a poor metabolic effect of insulin leading to chronic hyperglycaemia, which can result in microvascular complications. It is widely known that postprandial glycaemia is reliant on the total carbohydrate content of a meal. However, the importance of the amount and the source of these carbohydrates remains controversial due to mechanisms other than insulin secretion. Oxidative stress, inflammation, pyruvate production and the quality of the intestinal microbiota, resulting in plasma lipopolysaccharides and short-chain fatty acids production, play an important role in blood sugar control and consequently in type 2 diabetes. Thus, we systematically reviewed the preclinical evidences on the impact of the amount and type of carbohydrate found in different diets and its influence on blood glucose levels in diabetic animals. We used a comprehensive and structured search in biomedical databases Medline (PubMed), Scopus and Web of Science, recovering and analyzing 27 original studies. Results showed that sucrose-rich diets deteriorated diabetic condition in animal models regardless of the total dietary carbohydrate content. On the other hand, fiber, particularly resistant starch, improved blood glucose parameters through direct and indirect mechanisms, such as delayed gastric emptying and improved gut microbiota. All studies used rodents as animal models and male animals were preferred over females. Improvements in T2DM parameters in animal models were more closely related to the type of dietary carbohydrate than to its content on a diet, i. e., resistant starch seems to be the most beneficial source for maintaining normoglycemia. Results show that current literature is at high risk of bias due to neglecting experimental methods.

## Introduction

Diabetes mellitus has become one of the most common chronic diseases in the world, with 422 million people affected worldwide. Approximately 95% of people currently diagnosed with diabetes have type 2 diabetes mellitus (T2DM) [1]. The disease is marked by chronic hyperglycemia, which can impair pancreatic beta cell function and increase insulin resistance, deteriorating the metabolic condition [2] and causing microvascular complications in the retina, kidney or peripheral nerves [3]. Thus, glycemic control is essential for diabetes management, in order to avoid complications in organs and systems, which are related with high morbidity and mortality rates [1, 4].

Among T2DM causes are genetic and epigenetic elements interacting within a societal framework [5]. The genetic predisposition for T2DM takes into account the increased risk of an individual to develop T2DM when there are other family members affected [5]. On the other hand, epigenetic elements are those influenced by environmental factors, i. e., they can be reversible, and therefore manipulated, in order to treat the disease [6, 7]. Regardless of the causes, the main targets of the treatment are focused on decreasing insulin resistance and improving beta cell function through diet, exercise and oral hypoglycemic and anti-hyperglycemic agents [7, 8]. Based on this, given the high prevalence of the disease and the significant benefits of its management, it is of clinical importance to determine the proper amount and type of carbohydrates in patients’ diet, as they both may influence the glycemic index of a meal [9]. Besides, some foods’ intake induce a marked rise followed by a more or less rapid fall in blood glucose, while others produce a smaller peak along with a more gradual decline in plasma glucose [10]. Currently, it is recommended for T2DM patients an intake of 26–44% of total daily energy from carbohydrates, preferably from high-quality sources, such as vegetables, whole fruits and legumes [11], which are rich in fiber. Most fibers, which are unabsorbed carbohydrates, are insoluble and increase stool weight [12]. In contrast, starches are carbohydrates found in vegetables that are mostly broken down to sugars by digestive enzymes [12]. Resistant starch, also found in vegetables and whole fruits, escapes digestion, being fermented in the intestine as well as dietary fiber [12].

Although the quantity and the quality of carbohydrates in the diet influence blood glucose levels [13], influencing insulin secretion and gastric emptying [13], the most beneficial type, the ideal amount of dietary carbohydrates and the underlying mechanisms involved remain a matter of debate. Thus, this study was designed to systematically review the *in vivo* preclinical effects of the type and amount of dietary carbohydrates in studies involving T2DM animal models, in order to clarify these aspects for improving T2DM management. Furthermore, this review also evaluated the methodological quality of current evidence, pointing out the main sources of bias in the selected studies.

## Methods

### Focus question

The main question to be answered in this systematic review was: what are the ideal type and amount of dietary carbohydrate in order to improve T2DM parameters in animal models, and what are the main mechanisms involved in it?

### Literature search

This systematic review adhered to the PRISMA guidelines [14] (Preferred Reporting Items for Systematic Reviews and Meta-Analysis), including search strategy, selection criteria, extraction and data analysis. We completed a comprehensive bibliographic search using the electronic databases Pubmed/Medline (https://www.ncbi.nlm.nih.gov/pubmed), Scopus (https://www.scopus.com/home.uri) and Web of Science (https://www.webofknowledge.com). The studies were selected through an advanced search on the platforms Medline, Web of Science and Scopus, on the 10^th^ of January, 2020 at 1h10 pm. Based on two search parameters, we devised a comprehensive search strategy for the retrieval of all relevant studies: (i) direct searches in electronic databases, and (ii) indirect screening of reference lists from all studies identified in the direct searches. The keywords for filters for three criteria were type 2 diabetes mellitus, dietary carbohydrates and animal studies (S1 Table). The search filter for PubMed/Medline was based on standardized descriptors obtained from the hierarchical *Thesaurus* MeSH (Medical Subject Headings. In PubMed/Medline, the commands MeSH and TIAB (title and abstract) were combined for the retrieval of indexed papers and those citations in the indexing process (*epub ahead of print*). The same research descriptors were structured according to the specific search algorithms required in Web of Science (TS = *descriptor*) and Scopus (TITLE-ABS-KEY[*descriptor*]) databases [15]. No chronological limits were applied in the primary search. All original full-text studies published up to 2020 were included in the systematic review. The search strategy is detailed in the S1 Table.

Two reviewers (AMM and BSL) conducted the literature search, removed duplicate articles, and screened titles and abstracts with respect to eligibility criteria. After initial screening, full-text articles of potentially relevant studies were independently assessed for eligibility by two reviewers (MMS and MBF). The kappa test was used for the selection and data extraction (kappa = 0.946). Selections were then compared and inconsistencies were resolved in consultation with two other reviewers (RDN and RVG).

### Study selection

To discard the subjectivity in data collection and selection process, the information was independently extracted by two reviewers (RDN and RVG) and analyzed separately.

We retrieved only experimental original studies performed *in vivo*, developed with animal models, published in English and with full texts available. We selected only studies that met all of the eligibility criteria listed below:

- Studies including glycemic control parameters;
- Studies targeting type 2 diabetic animals;
- Studies reporting the effects of different dietary carbohydrate content or different types of dietary carbohydrates on T2DM.

**Fig 1:**
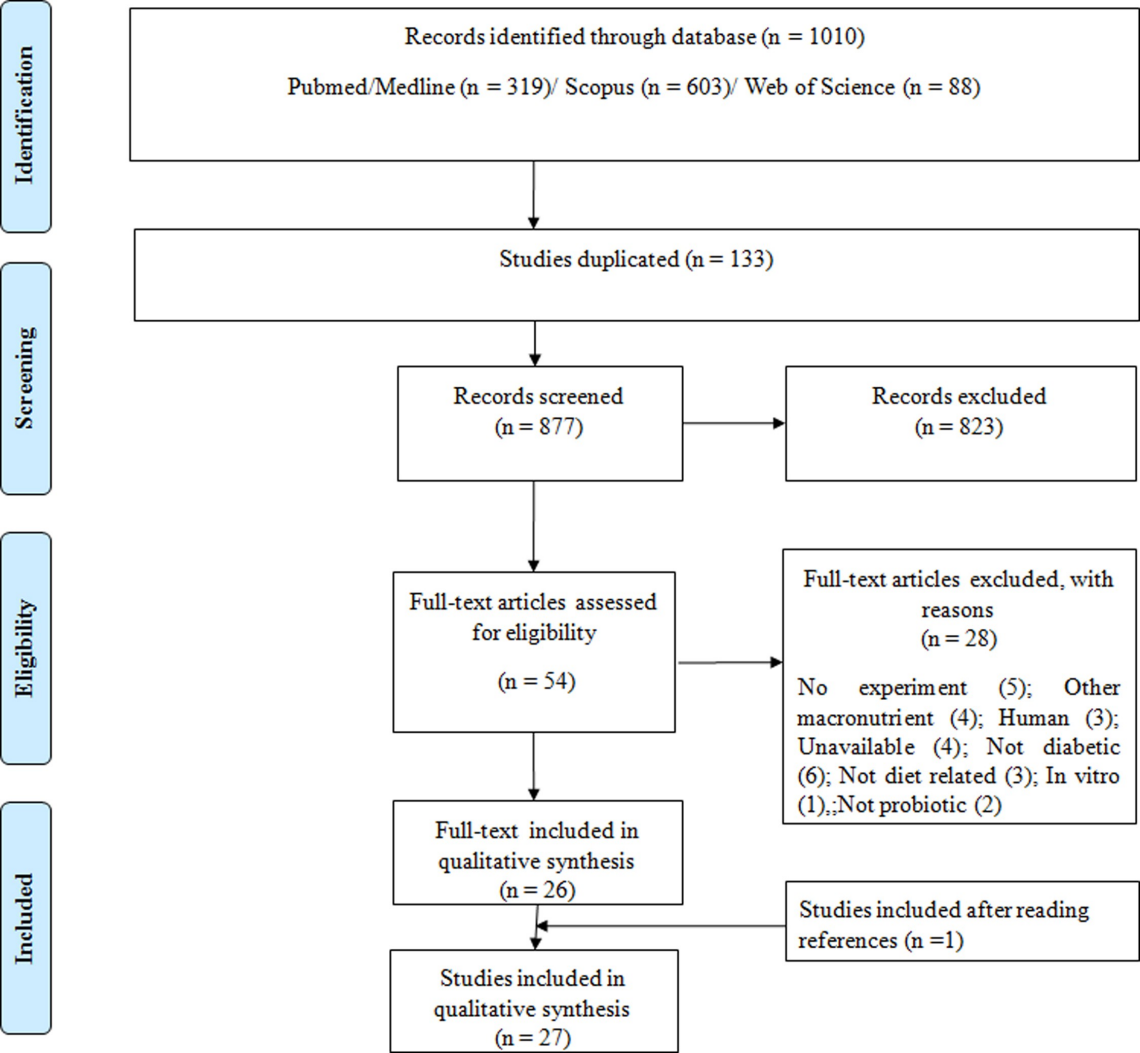
Flowchart detailing selection of studies included in systematic review. Based on PRISMA statement “Preferred Reporting Items for Systematic Reviews and Meta-Analyses”. www.prisma-statement.org.

The exclusion criteria were: (i) papers with no full-text available; (ii) secondary studies (i.e., literature reviews, comments, letters to the editor and editorials); (iii) grey literature (studies not peer-reviewed or formally published in indexed journals). The flowchart indicating the process of study selection is presented in Fig 1.

### Data extraction

Considering a comprehensive description of the research models, data extraction was based on methodological features and the studies were synthesized admitting different descriptive levels as it follows: (1) Publication characteristics (authors, year, country of origin); (2) characteristics of the animal models: species, strain, number of animals, sex, age/weight, intervention, total time of experiment); (3) performed analyses, primary findings and secondary findings. In the absence of available data within the study, authors were contacted via e-mail to provide further information.

Studies were initially grouped into diet categories based on the degree of carbohydrate restriction of the intervention diet according to the parameters established by Sainsbury *et al*. (2018) [16]. A low carbohydrate diet was defined as <26% of total energy from carbohydrate per day. Moderate carbohydrate diets were defined as between 26% and 45% of total energy from carbohydrate per day. High carbohydrate diets were those >45% of total energy from carbohydrates per day [16]. Due to wide variations within high-carbohydrate diets in regards to carbohydrate amounts, we added a new cathegory, very-high-carbohydrate diets, considering those with >70% of total energy per day from carbohydrates. Studies were also divided according to the type of carbohydrate (sucrose, glucose, fructose, fiber, resistant starch) and glycaemic index, when available. Data were subsequently compared and conflicting information was identified and corrected after discussion among the reviewers [17].

### Quality assessment

We assessed study quality through SYRCLE's risk of bias tool for animal studies (Systematic Review Centre for Laboratory animal) [18], adapted from Cochrane Collaboration. The assessment was made based on the following topics: 1. Random sequence generation, 2. Allocation concealment, 3. Blinding of caregivers and/or investigators from knowledge regarding interventions each animal received, 4. Blinding of outcome assessment, 5. Incomplete outcome data and 6. Selective outcome reporting [18]. The items in the RoB tool were scored with “yes” (low risk of bias), “no” (high risk of bias); or “unclear” (indicating that the item was not reported and therefore the risk of bias was unknown). Two review authors (AMM and BSL) independently assessed the risk of bias for each study using the adapted criteria outlined in the Cochrane Handbook for Systematic Reviews of Interventions. Any disagreements were resolved by discussion between authors. The risk of bias figures were created using Review Manager 5.3 [19].

## Results

### Characteristics of publications

**Fig 2:**
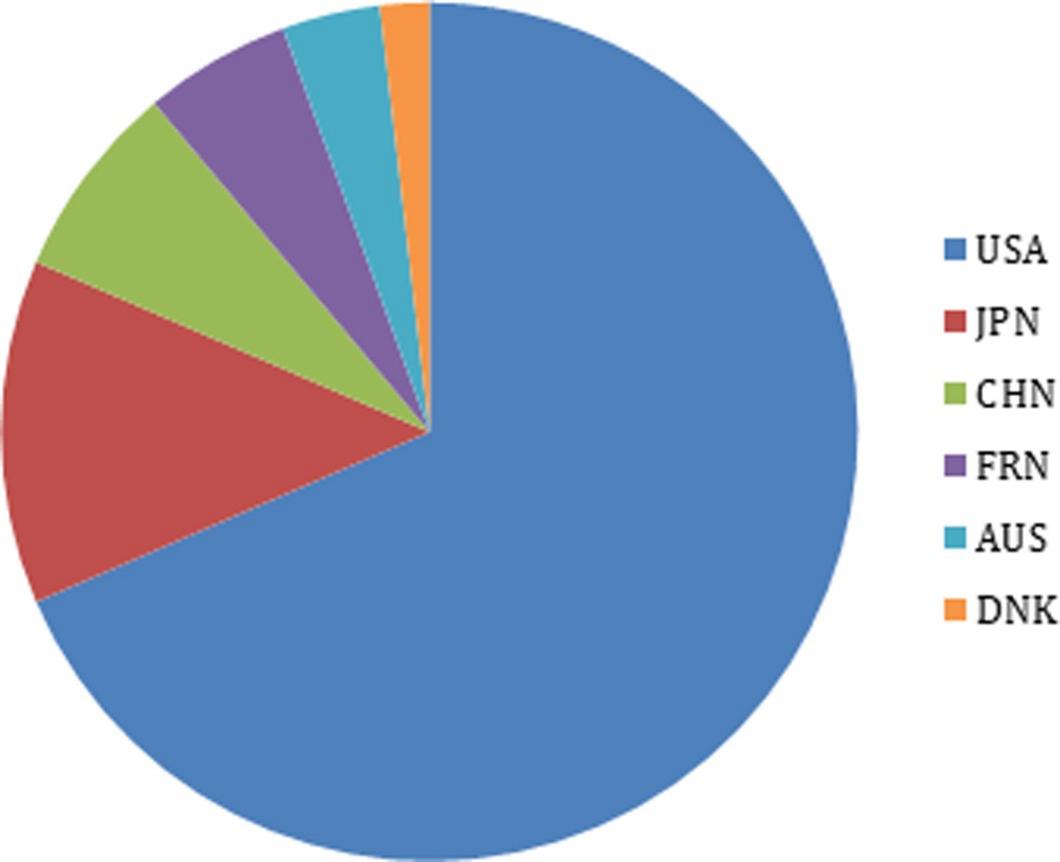
Country of origin of the studies included in this review. USA = United States of America; JPN = Japan; CHN = China; FRN = France; AUS = Australia; DNK = Denmark.

The initial search resulted in 1010 studies and 133 were duplicates. After reading the title and abstract, we excluded 823 studies that did not meet eligibility criteria. After this primary selection, the remaining 54 articles were completely read and 28 articles were excluded. The bibliographical references of the 26 selected articles were analyzed, and 1 study was added according to the inclusion criteria, resulting in 27 studies (Fig 1). Most studies identified originated from the United States of America (37%, n = 10), followed by Japan (26%, n = 7) and China (15%, n = 4). The remaining studies were from France (n = 3), Australia (n = 2) and Denmark (n = 1) (Fig 2). The country of origin of each study is found in Table 1.

**Table 1:** Qualitative description of main negative^‡^ and positive* outcomes reported in all studies investigating the relevance of types and amount of carboydrates used in T2DM diets in animal models.

Study/Country	Dietary strategy	Feed manufacturer	Negative outcomes‡
**Very High carbohydrate diet**	** **		** **
Parkman *et al*., 2016 [30] USA	70,8% CHO: sucrose	Purina 5001, BMI Nutrition, Brentwood, USA	↓glucose tolerance ↑BW
Arimura *et al*., 2017 [31] JPN	71% CHO 59% CHO high protein	Wako Pure Chemical Industries, JPN	↑BG in both diets albumin excretion higher in High protein group No difference in BW or C-Peptide
Arimura *et al*., 2018 [32] JPN	71% CHO, 12% Protein 59% CHO, 24% Protein	-	High protein diet ↑HbA1c, ↑plasma insulin and retinal thickness No difference in BG, urinary glucose and BW
**Author/Year/Country**	**Dietary strategy**	**Feed manufacturer**	**Negative outcomes**^**‡**^
**Very High carbohydrate High fiber diet**	** **		** **
Bolsinger *et al*., 2013 [29] USA	HC: 70% CHO MC: 40% CHO LC: 10% CHO HC+High Fiber: 70% CHO	-	Fasting BG higher in HC, followed by MC HC+High Fibre showed same results as LC
**Study/Country**	**Dietary strategy**	**Feed manufacturer**	**Negative outcomes**^**‡**^
**High carbohydrate diet**	** **		** **
Bhathena *et al*., 1989 [23] USA	54% CHO: Sucrose or starch	Teklad test diet, Madison, USA #40060	↑BG in sucrose group compared to starch ↑TC, TG and BW in the sucrose-fed group
Velasquez *et al*., 1995 [24] USA	54% CHO: Sucrose or starch	Teklad test diet, Madison, USA #40060	↑urinary glucose, ↑BG Sucrose-fed: ↑protein excretion, abnormal glomeruli and ↑plasma insulin
Kazumi *et al*., 1997 [25] JPN	Chow + 10% glucose or fructose in water	CE-2, Oriental Yeast, Tokyo, JPN	Both glucose and fructose ↑BG Fructose ↑TG
Patel *et al*., 2009 [26] AUS	61% CHO: fructose or cornstarch	-	Fructose: ↑fasting BG and ↓glucose tolerance ↑arterial stiffness
Nojima *et al*., 2013 [27] JPN	47,8% CHO: 30% sucrose or 50% fat	CRF-1 Oriental Yeast, Tokyo, JPN	Sucrose-fed: ↓glucose tolerance and ↑BW Fat-fed: ↑BG
Zhuo *et al*., 2018 [28] CHN	61% CHO: sucrose	-	↑fasting BG, insulin, TC, TG, GLUT4 ↓GLUT2 in the liver
**Author/Year/Country**	**Dietary strategy**	**Feed manufacturer**	**Negative outcomes**^**‡**^
**Moderate carbohydrate diet**	** **		** **
Noonan & Banks, 2000 [40] USA	35% CHO: sucrose	F2685, Bioserv Frenchtown, USA	↑fasting BG, BW and plasma insulin
Iwama *et al*., 2003 [20] JPN	30% CHO: sucrose	-	↑fasting BG, necrosis in pulpal tissue and alveolar bone reabsorption
**Author/Year/Country**	**Dietary strategy**	**Feed Manufacturer**	**Negative outcomes**^**‡**^
**Low carbohydrate diet**			** **
Pascoe *et al*., 1992 [33] AUS	20% CHO	Allied Feeds, Sydney, Australia	↑BG, BW, TC
Surwit *et al*., 1995 [34] USA	25% CHO (High fat): HSHFD, LSHFD	Research Diets, New Brunswick, USA	High fat diet ↑BG, BW and plasma insulin (both HSHFD and LSHFD)
Kaneko *et al*., 2000 [35] JPN	40% CHO 20%CHO	CE-2 Nippon Clea, Tokyo, Japan	20% and 40% CHO: ↑fasting BG and ↓glucose tolerance 20% CHO: ↑BW and plasma insulin
Wang *et al*., 2003 [36] JPN	10% CHO, 65% Fat	-	10% CHO: ↑fasting BG, ↑BW, ↓plasma insulin and ↓glucose tolerance
Petro *et al*., 2004 [37] USA	26% CHO	Research Diets, New Brunswick, USA	↑fasting BG, BW and plasma insulin
Asghar *et al*., 2006 [38] CHN	12% CHO: sucrose	Research Diets, New Brunswick, USA	↑fasting BG ↑glucagon ↑plasma insulin
**Study/Country**	**Dietary strategy**	**Feed Manufacturer**	Positive outcomes*
**Very High carbohydrate High fiber diet**	** **		** **
Zhou *et al*., 2015 [22] CHN	80% CHO: resistant starch	-	↓BG, TC and TG ↑BW
**High carbohydrate High fiber diet**	** **		** **
Hedemann *et al*., 2017 [21] DNK	52,95% CHO: Cornstarch, GLU, EMS orresistant starch	Altromin 1321, Brogaarden, DNK	↓Fasting BG in resistant starch and cornstarch-fed ↓HbA1c in resistance starch-fed All diets ↑TG
**Study/Country**	**Dietary strategy**	**Feed Manufacturer**	Positive outcomes*
**Moderate carbohydrate High fiber diet**	** **		** **
Shen *et al*., 2011 [39] USA	30% CHO: resistant starch	National Starch Food Innovation, Bridgewater, USA	↓fasting BG, ↑insulin sensitivity, ↑cecal short chain fatty acids and butyrate producing bacteria
**Study/Country**	**Dietary strategy**	**Feed Manufacturer**	Positive outcomes*
**Low carbohydrate High fiber diet**	** **		** **
Marsh et al., 2009 [46] USA	2% CHO	TestDiet, Richmond, USA	↓Fasting BG and HbA1c ↑arterial stiffness
Sun et al., 2018 [45] CHN	Resistant starch: Low dose (10%) Medium dose (15%) High dose (20%)	National Starch and Chemical Company, Shanghai, CHN	↓fasting BG, TC, TG and BW ↑plasma insulin

USA = United States of America; BG = blood glucose; TC = total cholesterol; TG = triglycerides; BW = body weight; CHO = carbohydrate; JPN = Japan; AUS, = Australia; HC = high carbohydrate; MC = moderate carbohydrate; LC = low carbohydrate; CHN = China; HbA1c = glycated hemoglobin A1c;— = missing info; HSHFD = high sucrose high fat diet; LSHFD = low sucrose high fat diet; LSLFD = low sucrose low fat diet; HSLFD = high sucrose low fat diet; DNK = Denmark; ♀ = female; GLU = glucidex; EMS = enzimatically modified starch; IHC = immunohistochemestry.

### Characteristics of experimental animals

All studies reported the use of rodents as experimental models, being 63% (n = 17) *Rattus novergicus*, 33% (n = 9) *Mus musculus* and 4% (n = 1) *Arvicanthis niloticus*. Male animals (96%, n = 26) were preferred over females (4%, n = 1). From the studies using *Rattus novergicus*, Wistar was the strain of choice (35%, n = 6), followed by Sprague-Dawley (29%, n = 5), and the age of experimental animals varied from 3 to 18 weeks. From the studies using *Mus musculus*, the strain of choice was C57BLKS (25%, n = 3) and the age of experimental animals varied from 3 to 5 weeks. For those using *Arvicanthis niloticus*, the strain of choice was the Nile rat (100%, n = 1) and the age for animals used in the study was 4 weeks. Three studies did not report the age of the experimental models [20–22]. Further details are found in S2 Table

### Characteristics of dietary strategies

Most studies used high carbohydrate diets (48%, n = 13), from which 15% (n = 2) were high carbohydrate and high fiber diets. Low carbohydrate diets accounted for 30% of the studies (n = 8), from which 13% (n = 1) were low carbohydrate and high fiber diets. Diets with moderate amounts of carbohydrates accounted for 11% of the studies (n = 3), from which 34% (n = 1) were moderate carbohydrate and high fiber diets. Very high carbohydrate diets accounted for 15% (n = 4) of the studies, from which 25% (n = 1) were very high carbohydrate and high fiber diets. Sucrose was the main carbohydrate source reported on these articles, representing 30% (n = 8) of the studied diets. Other carbohydrate sources reported in the selected studies were resistant starch (n = 4, 15%), glucose and fructose (n = 3, 11%). High glycaemic index diets were reported in 2,7% of the studies. The type of carbohydrate was not reported in 37% of the studies (n = 10) and only the percentage of dietary carbohydrates was evaluated in these studies. Most studies (59%, n = 16) purchased their diets from feed manufacturers, from which the nutritional composition was approximately 23% crude protein, 4.5% crude fat, 6% crude fiber, 8% ash, 2.5% added minerals; provided it *ad libitum*. Further details are found in Table 1.

### Main outcomes

Eighteen studies reported a worsening in blood glucose parameters; six of them intervened with a high carbohydrate diet [23–28]. One study intervened with a very high carbohydrate high fiber diet [29] and 3 studies intervened with a very high carbohydrate diet [30–32]. Six articles reported worsened blood glucose parameters under low carbohydrate diets [33–38] and 2 under moderate carbohydrate diets [39, 40]. Most diets that deteriorated blood glucose parameters were rich in sucrose (n = 8). Four studies with high dietary carbohydrate content reported no differences in glycemia in animals fed with different types of carbohydrates (corn starch on a high glycemic index diet, glucose and fructose) [41–44]. One study improved blood glucose parameters combining a low carbohydrate diet and resistant starch as the carbohydrate type (low carbohydrate high fiber diet) [45] and one study improved T2DM parameters on a low carbohydrate high fiber diet [46]. Detailed analyzes of diets, carbohydrates type, experimental models and outcomes are found in Tables 1 and 2).

**Table 2:** Summary of the impact of different types of diets on main parameters of T2DM in animal models.

Carbohydrate type	Diet^a^	Effect
Sucrose (n = 8)	VHC (n = 1)	Worsened plasma blood glucose *vs*. control group (n = 8)
	HC (n = 4)	
	MC (n = 2)	
	LC (n = 1)	
Glu/Fru (n = 3)	HC (n = 3)	Worsened plasma blood glucose *vs*. control group (n = 2) No difference (n = 1)
	MC (n = 0)	
	LC (n = 0)	
Corn starch (HGI diet) (n = 2)	HC (n = 2)	No difference (n = 2)
	MC (n = 0)	
	LC (n = 0)	
Resistant Starch (n = 4)	VHC + high fiber (n = 1) HC + high fiber (n = 1)	Improved plasma blood glucose *vs*. control group (n = 4)
	MC + high fiber (n = 1)	
	LC + high fiber (n = 1)	
NP (n = 10)	VHC (n = 2)	Worsened plasma blood glucose *vs*. control group (n = 8) Improved plasma blood glucose *vs*. control group (n = 1) No difference (n = 1)
	HC (n = 1)	
	HC + high fiber (n = 1)	
	MC (n = 0)	
	LC (n = 6)	

VHC = very high carbohydrate; HC = high carbohydrate; MC = moderate carbohydrate; LC = low carbohydrate; BG = blood glucose; HbA1c = glycated hemoglobin A1c; Glu/Fru = glucose and fructose; HGI = high glycemic index; NP = not provided.

^a^As established by Sainsbury *et al*. (2018) [14].

### Secondary outcomes

In regards to secondary results, most frequent parameters reported were body weight and plasma insulin concentration (both 74% of the studies); followed by plasma triglycerides (37% of studies) and total cholesterol concentrations (44% of studies), all these related to sucrose intake. High carbohydrate diets increased body weight in all studies, particularly when the main carbohydrate source was sucrose. In the absence of fiber, both low-carbohydrate diets [34–38] and high-carbohydrate diets [24, 28, 31, 32] led to increased insulin secretion. Similarly, both low- [35, 36] and high-carbohydrate diets [26, 27, 30] resulted in decreased glucose tolerance when no fiber was available in the diet.

Lipid profile was improved by carbohydrate-rich diets, as longs as the main carbohydrate source was resistant starch [23, 33]. Ten out of the 27 selected studies reported an increase in plasma insulin and 40% of these intervened with sucrose as a carbohydrate source, with content varying between 12–61%.

All studies used fasting blood glucose, blood glucose, urinary glucose, HbA1c (hemoglobin A1c), fructosamine, oral GTT (glucose tolerance test), intraperitoneal GTT or a combination of these as parameters to monitor T2DM. In addition, most articles reported effects on body weight, blood insulin and lipid profile.

The main mechanisms involved with carbohydrate intake that impair T2DM parameters were increased pyruvate production leading to fatty liver and increased serum lipids leading to metabolic syndrome [23, 24, 26–30, 33–38, 40]. Three studies reported a worsening in T2DM parameters unrelated to these mechanisms [20, 31, 32]. Improvements in T2DM parameters were associated with prebiotic effects of fiber and resistant starch, increased satiety and increased gastrointestinal transit time [21, 22, 39, 45]. One study reported improved glycemic parameters without further analysis of the underlying mechanisms [46].

### Quality assessment

**Fig 3:**
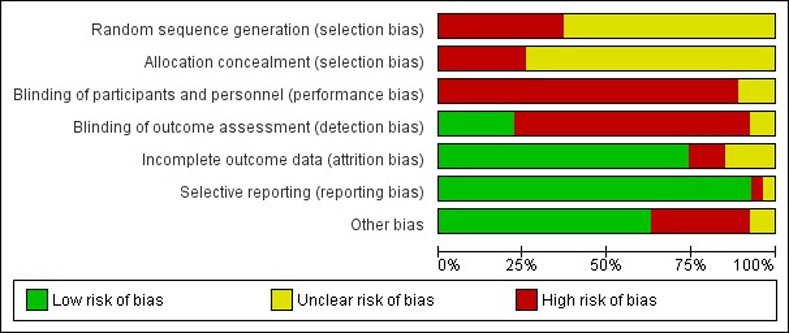
Risk of bias showing review authors' judgement about each risk of bias item presented as percentages across all included studies. The following methodological domains based on RoB were evaluated. Consider selection bias: “Was the allocation sequence adequately generated and applied?”, “Were the groups similar at baseline or were they adjusted for confounders in the analysis?”, “Was the allocation to the different groups adequately concealed?”; Consider performance bias: “Were the animals randomly housed during the experiment?”, “Were the caregivers and/or investigators blinded from knowledge regarding which intervention each animal received during the experiment?”; Consider detection bias: “Were animals selected at random for outcome assessment?”, “Was the outcome assessor blinded?”; Considers attrition bias: “Were incomplete outcome data adequately addressed?”; Considers reporting bias: “Are reports of the study free of selective outcome reporting?”; Considers other biases: “Was the study apparently free of other problems that could result in high risk of bias?”; The overall study quality indicators: “Was it stated that the experiment was randomized at any level?” and “Was it stated that the experiment was blinded at any level?”. The items in the RoB tool were scored with “yes” (low risk of bias); “no” (high risk of bias); or “unclear” (indicating that the item was not reported, and therefore, the risk of bias was unknown) [12]. The items in the RoB tool were scored with “yes” (low risk of bias); “no” (high risk of bias); or “unclear” (indicating that the item was not reported, and therefore, the risk of bias was unknown) [17].

**Fig 4:**
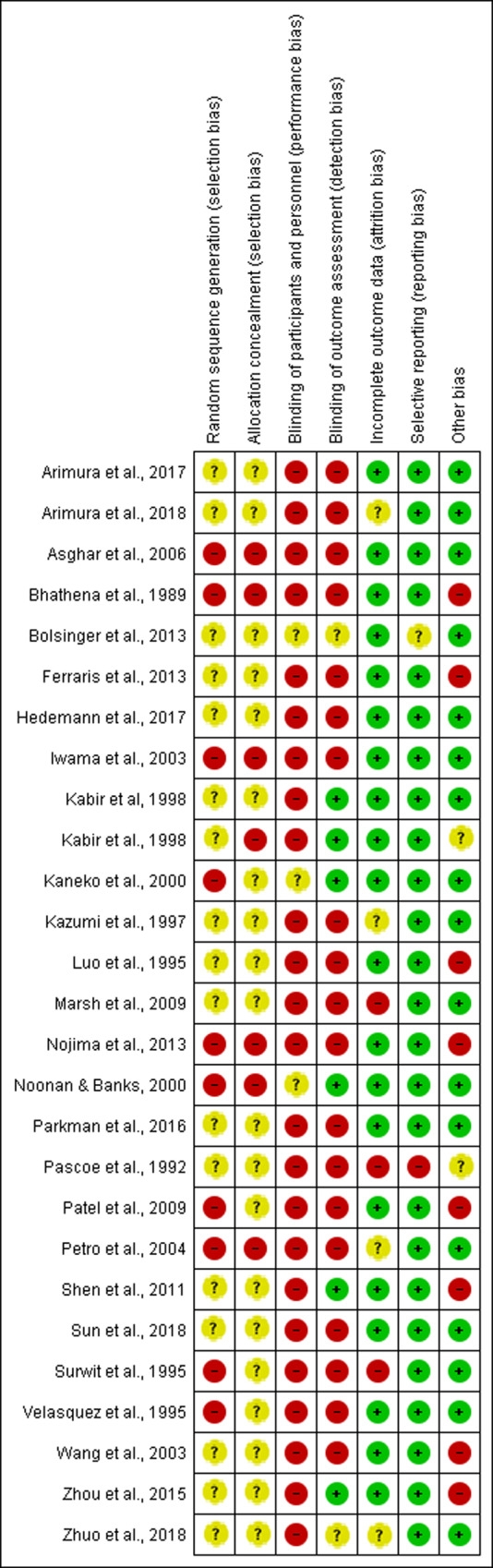
Risk of bias summary showing studies quality assessment at an individual level.

Overall, the assessment of key quality indicators resulted in high risk of bias for the selected studies. Most studies (62%, n = 17) reported that animals were randomly allocated without providing further details on how allocation was designed, resulting in unclear risk of bias. The remaining 38% of the studies did not report information on random allocation, which resulted in high risk of bias. Blinding among groups was under-reported and resulted in high risk of bias in 93% of the studies (n = 25). Only 6 studies (22%) reported blinding of personnel. Incomplete outcome data was adequately addressed in 85% of the studies (n = 23) and most studies were free from selective reporting (n = 25). Selected studies that are apparently free from other problems that could result in a high risk of bias accounted for 62%. Only 3 studies (11%) provided a conflict of interest statement and 24 studies (71%) mentioned approval by an ethical board. An overview of the main results of included articles was schematically shown in Fig 3. Quality assessment at an individual level was reported in Fig 4.

## Discussion

In our study, we conducted a systematic review to investigate the effect of the amount and the type of carbohydrates used in diets for T2DM animal models. Our results showed that sucrose deteriorates blood glucose levels regardless of its dietary content. On the other hand, resistant starch and dietary fiber seem to improve T2DM parameters even when associated with high-carbohydrate diets. Besides, our findings show that the studies investigating carbohydrate intake are concentrated in the United States, Japan and China. As the staple food in these countries is carbohydrate-based, rich in wheat and/or rice, the increased concentration of studies in these countries may be related to concerns on their population eating habits, in addition to the fast increase of fast food restaurants available worldwide. In The United States, convenience and/or high sugar foods are largely available in a wide variety of formats, which contributes to poor dietary habits. In addition, large portion sizes, usually offered in restaurants, also raise concern on the amount of extra calories ingested [47]. In Asian countries, a “nutrition transition” from a traditional, vegetable-based diet to a processed, unhealthy diet has been occurring along younger generations. This change has been related to the rapid growth of non-communicable diseases in China and Japan, such as diabetes [48].

Although our search was not limited to rodents, all experimental models used in the selected articles were murines, which exclude other potentially useful animal models. While humans are undoubtedly the model of choice when studying the pathophysiology of human disease, using living humans as experimental models has several logistical and ethical limitations. Therefore, there is a need to develop *in vivo* animal models for this purpose. Frequently used genetic models of T2DM, such as db/db mice and Zucker fa/fa rats, have been useful in understanding mechanisms which contribute to disease development, however, they are not ideal models as these gene mutations are extremely rare in human populations [49]. Similarly, T2DM murine models induced by destruction or pancreatic ablation [50] are not representative of the etiology of T2DM in humans. As T2DM is linked to excessive accumulation of body fat, diet-induced obesity models are particularly relevant for investigating underlying mechanisms through which an excessive dietary fat and/or sugar intake may result in insulin resistance and the onset of T2DM. Preclinical studies have shown that overfeeding may induce obesity, low grade inflammation and insulin resistance in 8 to 80 weeks [51, 52].

Nutritional manipulations to induce T2DM in animals include changing the diet itself or maternal diet during pregnancy and/or lactation; and involve either increases in dietary fat or carbohydrates. The study of diet-induced obesity and models of prenatal undernutrition and overnutrition has revealed several common mechanisms that contribute to the understanding of the physiological basis of reduced insulin sensitivity and provide some new insights into T2DM etiology in humans [49]. A low birth weight followed by a period of increased postnatal growth, or a high birth weight due to prenatal overnutrition, are both associated with a higher chance towards developing insulin resistance, glucose intolerance, and T2DM in adult life [53]. The two most widely used models for the study of T2DM found in this review were high-fat and high- sugar feeding in rodents. The high-fat feeding animal model C57BL/6 was the most used mice strain in the included studies, predominantly with *ad libitum* access to a high-energy diet from 2 to 16 weeks, developing glucose intolerance, obesity and hyperglycemia. Regarding the high-sugar feeding animal models, the most used strains were Wistar and Sprague-Dawley rats, fed *ad libitum* for 3–64 weeks, chosen for studying the metabolic effects of diet-induced obesity [49, 54], as shown by the results of our review. Considering the need to improve and standardize protocols of preclinical models for T2DM studies, it is important to highlight differences between commonly used rodent species. In rats, providing sucrose either in solid form or in drinking water (high-sugar diet) has been associated with both increased visceral fat accumulation and insulin resistance in both liver and skeletal muscle [55]. In mice, however, adding sucrose to drinking water fails to induce obesity, although this does lead to subtle metabolic changes, such as adipocyte hypertrophy, glucose intolerance, hyperinsulinemia, hyperlipidemia, fatty liver and increased levels of inflammatory cytokines [55]. Hence, further studies in the area of high-fat/high-sucrose feeding are required in order to establish the best model of T2DM and to completely elucidate the effects of the diet in this case.

Our results also show that most studies retrieved used only males as experimental models. Single sex studies still prevail in the biological literature [56], although studies limited to only one gender cannot yield a complete understanding of gender-related differences and the underlying mechanisms involved, even if they only occur in certain environments, at specific ages or stages of the reproductive cycle. A partial list of sexually dimorphic rodent behavioral traits included wheel running behavior, open field activity, aggression, taste preferences, food intake, performance on learning tasks and responses to brain damage. Animals in weaning age were more frequently used compared to older ones, which may be due to a better adaptation to the experimental diets [57]. Most diets (59%, n = 16) were acquired from feed manufacturers, which allows controlled methodological standards and improves studies’ reproducibility.

### Sucrose, fructose, glucose and high glycaemic index diets

In regards to diets, carbohydrates are the first macronutrient to be broken into glucose, which is the main insulin secretagogue. Thus, one can assume that simply decreasing carbohydrate intake would lead to improved diabetes management. However, each individual may respond differently to a variety of diets. Most HC diets containing mono- and disaccharides and no sources of fiber resulted in a deterioration of the diabetic condition, shown on fasting blood glucose tests, HbA1c, fructosamine, intraperitoneal GTT or oral GTT, regardless of the percentage of carbohydrates in the diet. At similar amounts of carbohydrates, low glycaemic index and high fiber meals tend to result in lower postprandial blood glucose compared to high glycaemic index meals [12, 58]. Controversially, the only 2 studies included in this review on high glycaemic index diets reported no differences in blood glucose parameters when animals were fed this type of diet. It has been suggested that an increase in GLUT4 at the cells membrane compensates for the high glycaemic index food [42]. It is known that a sucrose-rich diet can induce upregulation of GLUT5 in the apical border of enterocytes in the small intestine, which increases fructose absorption [59]. However, high fructose consumption can lead to excessive pyruvate production and enhanced lipid biosynthesis, as a consequence [60]. Hence, a sucrose rich diet could accelerate the development of metabolic syndrome and cause fatty liver disease. In addition, it may induce pancreatic inflammation with increased macrophage infiltration, which might reduce insulin secretion [61] and consequently blood glucose parameters’ deterioration. Similarly, diets containing only fructose and glucose showed worsened diabetes condition in 67% of the studies and the other 33% showed no difference. Only 3 studies used these carbohydrate types in their experimental diets from all studies included in this review. When the type of carbohydrate added was resistant starch, blood glucose parameters improved in all studies, regardless of the amount used.

Among the mechanisms that might be impairing well-managed diabetes is increased uric acid production, a product from sucrose and fructose metabolism due to the breakdown of adenine nucleotides [61]. Uric acid enters cells via specific transporters, such as URAT1, where it induces proinflammatory and prooxidative effects [62]. Sucrose-fed rats have been reported increased URAT1 expression in pacreatic islets, chronic hyperuricemia, hypertriglyceridemia and fatty liver [63], which supports the findings of the studies in this review.

In the same way, very high carbohydrate diets, in which dietary carbohydrate content was 70% or more of total daily energy intake, led to a pronounced deterioration of T2DM in animal models. Macronutrient composition modulates fatty acid deposition and inflammation in different tissues such as liver, brain and adipose tissue [64, 65]. In a study conducted by Antunes *et al*. [65], mice were fed a diet containing 73.8% carbohydrates for 2 months, resulting in increased lipid deposition and more intense inflammation due to increased proinflammatory prostaglandins and decreased anti-inflammatory mediators. This is in accordance with our findings, as both inflammation and lipid accumulation worsen metabolic syndrome [12], which is closely related to T2DM [1].

### Fiber

The American Diabetes Association (ADA) emphasizes that nutrition therapy is essential for T2DM patients [66], improving blood glucose levels and overall health. Currently, there is no ideal macronutrient proportions that applies broadly, thus, the dietary macronutrient ratio should be individualized. The recommended amount of carbohydrates for healthy adults is 130 g/day, determined considering mainly the brain’s requirement for glucose. However, this energy requirement is also fulfilled by other metabolic processes, as glycogenolysis, gluconeogenesis, and/or ketogenesis [67]. Currently, a common dietary intervention is the Mediterranean diet [68] that emphasizes plant-based foods, seafood and olive oil as the main source of dietary fat. It includes moderate amounts of dairy products, red meat and wine; and low or very low amounts of sugars. Benefits to T2DM patients include reduced HbA1c, lowered triglycerides and reduced risk of cardiovascular events [68]. The increasingly popular vegetarian and vegan dietary approaches emphasize plant-based eating and may include egg and/or dairy products (in case of vegetarian) or exclude all flesh foods and animal-derived products (in case of vegan) and both are shown to decrease HbA1c and body weight [69]. Low-fat diets emphasize vegetables, fruits, starches, lean protein sources and low-fat dairy products. Studies report weight loss as a common benefit for T2DM patients [70]. Low-carbohydrate diets emphasize vegetables that are low in carbohydrates and advise against starchy and sugary foods. Current diabetes reports consider 26–45% of total calories from carbohydrates as “low-carbohydrate” and fewer than 26% as “very low-carbohydrate” approach. These diets have been reported as a strategy for T2DM patients who are not reaching their glycemic goals with medication, as reported benefits include HbA1c reduction, weight loss, lowered blood pressure and lowered plasma triglycerides [71]. Another dietary strategy that has been used for T2DM management is the Paleo diet, which emphasizes foods eaten during early human evolution such as meat, fish, shellfish, vegetables and nuts. Benefits of this diet remain unclear due to inconclusive evidence [72].

Regardless of the type of diet ingested, increased fiber intake has been strongly recommended as part of T2DM management in humans due to its benefits in inducing satiety, increasing gastrointestinal transit time and improving overall blood glucose levels [73]. In addition, high-fiber diets are associated with lower all-cause mortality in people with T2DM [74], therefore, patients are encouraged to consume at least the amount of dietary fiber recommended for the general public (minimum of 14 g of fiber per 1,000 kcal) [66]. Thus, overall dietary recommendations for T2DM patients include avoiding added sugars and preferring carbohydrates from fiber-rich sources [66, 73, 74]. This supports our findings in animal models, that show that increased fiber intake and the use of resistant starch as a the main carbohydrate source has benefits for maintaining normoglycemia and overall health. Furthermore, added sucrose is not recommended in any of the abovementioned diets, which corroborates our findings that sucrose intake leads to a worsening in lipid profile, fatty liver disease, development of metabolic syndrome and T2DM onset.

### Resistance starch

**Fig 5:**
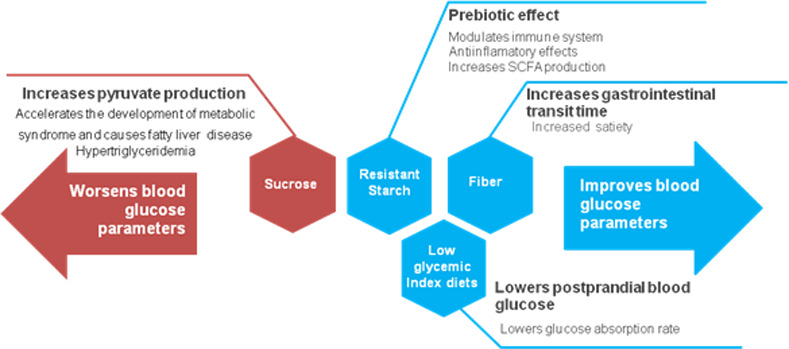
Effects of different sources of dietary carbohydrates on T2DM diets in metabolic parameters of animal models.

Regardless of the carbohydrate percentage in the diet, all studies using resistant starch included in this review resulted in an improvement of blood glucose parameters. As resistant starch may escape digestion, a diet rich in this particular fiber may be considered a carbohydrate-restricted diet. Resistant starch physically inaccessible to digestive enzymes is referred to as the type 1, found in whole grains and seeds [11]. Type 2 resistant starch is the one resistant to digestion due to the nature of the starch granule, found in raw potatoes and unripe bananas, for instance [11]. Types 3 and 4 result from food processing and chemical modification, respectively [11]. Due to its natural features and benefits, it has received a lot of attention as a functional ingredient, since resistant starch is fermented and used by the microbiota in the large intestine [75], resulting in beneficial bacterial growth, such as Lactobacilli, Bifidobacteriacea. Bacterial flora proliferation leads to increased SCFAs (short-chain fatty acids) production, which has anti-inflammatory properties. Therefore, resistant starch has been considered a prebiotic, being able to attenuate many metabolic disorders [76], through decreasing inflammatory status, increasing mucosal thickness and, as a result, reducing intestinal permeability to toxins. In addition, its presence can delay gastric emptying and the entrance of glucose into the bloodstream, decreasing postprandial glycaemia. It can also indirectly reduce insulin resistance and blood glucose levels due to reduced inflammation [75]. Different types of dietary carbohydrates on blood glucose parameters, considering the underlying mechanisms, are summarized in Fig 5.

### Fat

The National Academy of Medicine has defined that the acceptable macronutrient distribution for total fat for all adults is 20–35% of the total calorie intake [53]. Diets with high fat, low carbohydrate content have demonstrated important improvements in glycemia and cardiovascular risk factors compared with low fat, high carbohydrate diets. While there is an association between cholesterol intake and serum cholesterol levels, there is no direct link between cholesterol intake and cardiovascular events [69]. More research is needed on T2DM, serum cholesterol, cholesterol intake and cardiovascular events.

### Limitations

Systematic reviews are considered high-level studies that allow for the individual evaluation of studies in a blind manner using specific tools [77]. Such characteristics lead to a more inclusive and reliable approach, providing a broad understanding of the included studies. However, one limitation of our review is that studies were grouped into 4 degrees of carbohydrate intake, being: very-high carbohydrate diets (>70%), high carbohydrate (45–70%), moderate carbohyidrae diets (26–45%) and low carbohydrate diets (<26%). This could prevent definitive conclusions regarding the effect of carbohydrate amount in a diet as the range of carbohydrate intake is very wide among groups. Furthermore, commercial diets fed to control groups in most studies fit into the high carbohydrate diet group, which may hinder comparisons. Another limitation is that dietary carbohydrate type was neglected in some studies, preventing a deeper understanding of the role of carbohydrates on diets and the underlying mechanisms involved.

## Conclusion

Improvements in T2DM parameters in animal models were more closely related to the type of dietary carbohydrate than to its content on a diet, i. e., resistant starch seems to be the most beneficial source for maintaining normoglycemia and among the underlying mechanisms associated with it are decreasing inflammatory status, reducing intestinal permeability to toxins, delayed gastric emptying and delayed entrance of glucose into the bloodstream, decreasing postprandial glycaemia. Results also showed that current literature is at high risk of bias due to neglecting experimental methods.

## Supporting information

S1 Table Table of descriptors of research, containing the filters used in each one of the platforms. (DOCX)

S2 Table General characteristics of the experimental models used in all studies included in the systematic review. (DOCX)

S1 Checklist PRISMA 2009 checklist. (DOC)

S1 File (PDF)

## References

[1] Hu G, Weng J. Diabetes: leveraging the tipping point of the diabetes pandemic. Diabetes. 2017; 66(6):1461–1463. 10.2337/dbi16-0076 28533297

[2] Calero Bernal ML, Varela Aguilar JM. Infant-juvenile type 2 diabetes. Rev Clin Esp. 2018; 218(7):372–381. 10.1016/j.rceng.2018.03.015 29748149

[3] Egan MA, Dinneen SF. What is diabetes? Medicine. 2019; 47(1):1–4. 10.1016/j.mpmed.2018.10.002

[4] Diabetes Association of the Republic of China. Executive summary of the DAROC clinical practice guidelines for diabetes care– 2018. J Formos Med Assoc. 2019 Available online 3 April 2019. 10.1016/j.jfma.2019.02.016 30952480

[5] Meigs JB, Cupples LA, Wilson PW. Parental transmission of type 2 diabetes: the Framingham Offspring Study. Diabetes. 2000; 49: 2201–2207. 10.2337/diabetes.49.12.2201 11118026

[6] Wu C, Morris JR. Genes, genetics, and epigenetics: a correspondence. Science. 2001; 293: 1103–1105. 10.1126/science.293.5532.1103 11498582

[7] Lee YY, Park KS, Pak YK, Lee HK. The role of mitochondrial DNA in the development of type 2 diabetes caused by fetal malnutrition. J Nutr Biochem. 2005; 16: 195–204. 10.1016/j.jnutbio.2004.11.002 15808323

[8] Kim AY, Park YJ, Pan X, Shin KC, Kwak SH, Bassas AF, et al Obesity induced DNA hypermethylation of the adiponectin gene mediates insulin resistance. Nat Commun. 2015; 6: 7585 10.1038/ncomms8585 26139044 PMC4506505

[9] Abdelhafiz AH, Sinclair AJ. Diabetes, nutrition and exercise. Clin Geriatr Med. 2015; 31(3):439–451. 10.1016/j.cger.2015.04.011 26195102

[10] Kumar A, Sahoo U, Baisakha B, Okpani OA, Ngangkham U, Parameswarm C, et al Resistant starch could be decisive in determining the glycemic index of rice cultivars. J Cereal Sci. 2018; 79:348–353. 10.1016/j.jcs.2017.11.013

[11] Liebman M. When and why carbohydrate restriction can be a viable option. Nutrition. 2014; 30(7, 8):748–754. 10.1016/j.nut.2013.11.021 24984988

[12] Slavin JL. Carbohydrates, dietary fiber, and resistant starch in white vegetables: links to health outcomes. Adv Nutr. 2013;4(3):351S–5S. 10.3945/an.112.003491 23674804 PMC3650507

[13] Franz MJ, Bantle JP, Beebe CA, Brunzell JD, Chiasson JL, Garg A, et al Evidence based nutrition principles and recommendations for the treatment and prevention of diabetes and related complications. Diabetes Care. 2002; 25(1):148–198. 10.2337/diacare.25.1.148 11772915

[14] Moher D, Liberati A, Tetzlaff J, Altman DG. Preferred reporting items for systematic reviews and meta-analyses: the PRISMA statement. PloS Med. 2009;6(7). 10.1371/journal.pmed.1000097 19621072 PMC2707599

[15] Pereira RM, Greco GMZ, Moreira AM, Chagas PF, Caldas IS, Gonçalves RV, et al Applicability of plant-based products in the treatment of Trypanosoma cruzi and Trypanosoma brucei infections: a systematic review of preclinical in vivo evidence. Parasitology. 2017;144 (10):1275–1287. 10.1017/S0031182017000634 28578742

[16] Sainsbury E, Kizirian NV, Partridge SR, Gill T, Colagiuri S, Gibson AA. Effect of dietary carbohydrate restriction on glycemic control in adults with diabetes: A systematic review and meta-analysis. Diabetes Res Clin Pract. 2018; 139, 234–252. 10.1016/j.diabres.2018.02.026 29522789

[17] Altoé LS, Alves RS, Sarandy MM, Morais-Santos M, Novaes RD, Gonçalves RV. Does antibiotic use accelerate or retard cutaneous repair? A systematic review in animal models. PLOS ONE. 2019; 14(10), e0223511 10.1371/journal.pone.0223511 31600279 PMC6786583

[18] Hooijmans CR, Tillema A, Leenaars M, Ritskes-Hoitinga M. Enhancing search efficiency by means of a search filter for finding all studies on animal experimentation in PubMed. Lab Anim. 2010; 44, 170–175. 10.1258/la.2010.009117 20551243 PMC3104815

[19] Review Manager (RevMan) [Computer program] Version 5.3. Copenhagen: The Nordic Cochrane Centre, The Cochrane Collaboration, 2014.

[20] Iwama A, Nishigaki N, Nakamura K, Imaizumi I, Shibata N, Yamasaki M, et al The Effect of High Sugar Intake on the Development of Periradicular Lesions in Rats with Type 2 Diabetes. J Dent Res. 2003; 82(4):322–325. 10.1177/154405910308200416 12651940

[21] Hedemann MS, Hermansen K, Pedersen S, Bach Knudsen KE. Resistant Starch but Not Enzymatically Modified Waxy Maize Delays Development of Diabetes in Zucker Diabetic Fatty Rats. J Nutr. 2017; 147:825–834. 10.3945/jn.116.243899 28298535

[22] Zhou Z, Wang F, Ren X, Wang Y, Blanchar C. International Journal of Biological Macromolecules Resistant starch manipulated hyperglycemia/hyperlipidemia and related genes expression in diabetic rats. Int J Biol Macromol. 2015; 75:316–321. 10.1016/j.ijbiomac.2015.01.052 25661882

[23] Bhathena SJ, Kennedy BW, Jones J, Smith PM, Michaelis OE 4th, Carswell N, et al Effect of Dietary Carbohydrates on Insulin and Glucagon Receptorsin a New Model of Noninsulin Dependent SHR/N-corpulent Rat (42957). Proc Soc Exp Biol Med. 1989; 192(1):66–71. 10.3181/00379727-192-42957 2552455

[24] Velasquez MT, Abraham AA, Kimmel PL, Farkas-Szallasi T, Michaelis OE 4th. Diabetic glomerulopathy in the SHR/N-corpulent rat: role of dietary carbohydrate in a model of NIDDM. Diabetologia. 1995; 38(1):31–38. 10.1007/BF02369350 7744227

[25] Kazumi T. Effects of Dietary Fructose or Glucose on Triglyceride Production and Lipogenic Enzyme Activities in the Liver of Wistar Fatty Rats, an Animal Model of NIDDM. Endocrine Journal. 1997; 44(2):239–245. 10.1507/endocrj.44.239 9228459

[26] Patel J, Iyer A, & Brown L. Evaluation of the chronic complications of diabetes in a high fructose diet in rats. Indian J Biochem Biophys. 2009; 46:66–72. 19374256

[27] Nojima K, Sugimoto K, Ueda H, Babaya N, Ikegami H, Rakiji H. Analysis of hepatic gene expression profile in a spontaneous mouse model of type 2 diabetes under a high sucrose diet. Endocr J 2013; 60(3):261–274. 10.1507/endocrj.ej12-0258 23131898

[28] Zhuo J, Zeng Q, Cai D, Zeng X, Chen Y, Gan H, et al Evaluation of type 2 diabetic mellitus animal models via interactions between insulin and mitogen—activated protein kinase signaling pathways induced by a high fat and sugar diet and streptozotocin. Mol Med Rep. 2018; 17:5132–5142. 10.3892/mmr.2018.8504 29393432 PMC5865978

[29] Bolsinger J, Pronczuk A, Hayes KC. Dietary carbohydrate dictates development of Type 2 diabetes in the Nile rat. J Nutr Biochem. 2013; 24(11):1945–1952. 10.1016/j.jnutbio.2013.06.004 24070602

[30] Parkman JK, Mao X, Dillon K, Gudivada A, Moustaid-Moussa N, Saxton AM, et al Genotype-dependent Metabolic Responses to Semi- Purified High-Sucrose High-Fat Diets in the TALLYHO/Jng vs. C57BL/6 Mouse during the Development of Obesity and Type 2 Diabetes. Exp Clin Endocrinol Diabetes. 2015; 124(10):622–629. 10.1055/s-0042-109605 PMC11015344 27437918

[31] Arimura EM, Okatani H, Araki T, Ushikai M, Nakakuma M, Abe M, et al Effects of Diets with Different Proportions of Protein/Carbohydrate on Retinal Manifestations in db Mice. In vivo. 2018; 32(2):265–272. 10.21873/invivo.11233 29475908 PMC5905193

[32] Arimura E, Pralampita Pulong W, Marchianti ACN, Nakakuma M, Abe M, Ushikai M, et al Deteriorated glucose metabolism with a high - protein, low - carbohydrate diet in db mice, an animal model of type 2 diabetes, might be caused by insufficient insulin secretion. Eur Journal Nutr. 2017; 56(1):237–246. 10.1007/s00394-015-1075-y 26497335

[33] Pascoe WS, Jenkins AB, Kusunoki M, Storlien LH. Insulin action and determinants of glycaemia in a rat model of Type 2 (non-insulin-dependent) diabetes mellitus. Diabetologia. 1992; 35:208–215. 10.1007/BF00400919 1563580

[34] Surwit RS, Feinglos MN, Rodin J, Sutherland A, Petro AE, Opara EC, et al Differential Effects of Fat and Sucrose on the Development of Obesity and Diabetes in C57BL/6J and A/J Mice. Metabolism. 1995; 44(5):645–651. 10.1016/0026-0495(95)90123-x 7752914

[35] Kaneko T, Wang Y, Sato A. Low-carbohydrate / high-fat diet on the development of Diabetes mellitus in spontaneously diabetic rats. Diabetes Metab J. 2000; 26:459–464. 11173716

[36] Wang Y, Wang P, Qin L, Davaasambuu G, Kaneko T, Xu J, et al The Development of Diabetes Mellitus in Wistar Rats Kept on a High-Fat / Low-Carbohydrate Diet for Long Periods. Endocrine. 2003; 22(2):85–92. 10.1385/endo:22:2:85 14665711

[37] Petro AE, Cotter J, Cooper DA, Peters JC, Surwit SJ, Surwit RS. Fat, Carbohydrate, and Calories in the Development of Diabetes and Obesity in the C57BL/6J Mouse. Metabolism. 2004; 53(4):454–457. 10.1016/j.metabol.2003.11.018 15045691

[38] Asghar AZ. Insulin resistance causes increased beta-cell mass but defective glucose-stimulated insulin secretion in a murine model of type 2 diabetes. Diabetologia. 2006; 49:90–99. 10.1007/s00125-005-0045-y 16362284

[39] Shen L, Keenan MJ, Raggio A, Williams C, Martin RJ. Dietary-resistant starch improves maternal glycemic control in Goto–Kakizaki rat. Mol Nut Food Res. 2016; 55(10):1499–1508. 10.1002/mnfr.201000605 PMC4826617 21638778

[40] Noonan WT, Banks RO. Renal Function and Glucose Transport in Male and Female Mice with Diet-Induced Type II Diabetes Mellitus (44568), P.S.E.B.M. 2000; 225: 221–230. 10.1046/j.1525-1373.2000.22528.x 11082217

[41] Luo J, Rizkalla SW, Lerer-Metzger M, Boillot J, Ardeleanu A, Bruzzo F, et al A Fructose-Rich Diet Decreases Insulin-Stimulated Glucose Incorporation into Lipids but Not Glucose Transport in Adipocytes of Normal and Diabetic Rats. J Nutr. 1995; 125:164–171. 10.1093/jn/125.2.164 7861242

[42] Kabir M, Rizkalla SW, Champ M, Luo J, Boillot J, Bruzzo F, et al Dietary Amylose-Amylopectin Starch Content Affects Glucose and Lipid Metabolism in Adipocytes of Normal and Diabetic Rats. J Nutr. 1998; 128:35–42. 10.1093/jn/128.1.35 9430599

[43] Ferraris RP, Casirola DM, Vinnakota RR. Dietary Carbohydrate Enhances Intestinal Sugar Transport in Diabetic Mice. Diabetes. 1993; 42:1579–1587. 10.2337/diab.42.11.1579 8405698

[44] Kabir M, Rizkalla SW, Quignard-boulange A, Guerre-Millo M, Boillot J, Ardouin B, et al A High Glycemic Index Starch Diet Affects Lipid Storage–Related Enzymes in Normal and to a Lesser Extent in Diabetic Rats. J Nutr. 1998; 128:1878–1883. 10.1093/jn/128.11.1878 9808637

[45] Sun H, Ma X, Zhang S, Zhao D, Liu X. Resistant starch produces antidiabetic effects by enhancing glucose metabolism and ameliorating pancreatic dysfunction in type 2 diabetic rats. Int J Biol Macromol. 2018; 110:276–284. 10.1016/j.ijbiomac.2017.11.162 29191422

[46] Marsh SA, Italia LJ, Chatham JC. Interaction of diet and diabetes on cardiovascular function in rats. Am J Physiol Heart Circ Physiol. 2018; 296(2):1–21. 10.1152/ajpheart.00421.2008 19036853 PMC2643886

[47] Fulkerson JA. Fast food in the diet: Implications and solutions for families. Physiol Behav. 2018; 193(B):252–256. 10.1016/j.physbeh.2018.04.005 29630965

[48] Ma D, Sakai H, Wakabayashi C, Kwon JS, Lee Y, Liu S, et al The prevalence and risk factor control associated with noncommunicable diseases in China, Japan, and Korea. J Epidemiol. 2017; 27(12):568–573. 10.1016/j.je.2016.12.019 28623056 PMC5623033

[49] Wargent ET. Practical Considerations for In Vivo Mouse Studies. Methods Mol Biol. 2020; 2076:31–42. 10.1007/978-1-4939-9882-1_2 31586320

[50] Portha B, Blondel O, Serradas P, McEvoy R, Giroix MH, Kergoat M, et al The rat models of non-insulin dependent diabetes induced by neonatal streptozotocin. Diabete Metab. 1989; 15:61–75. 2525491

[51] Velázquez KT, Enos RT, Bader JE, Sougiannis AT, Carson MS, Chatzistamou I, et al Prolonged high-fat-diet feeding promotes non-alcoholic fatty liver disease and alters gut microbiota in mice. World J Hepatol. 2019 8; 11(8):619–637. 10.4254/wjh.v11.i8.619 31528245 PMC6717713

[52] Xu L, Nagata N, Chen G, Nagashima M, Zhuge G, Ni Y, et al Empagliflozin reverses obesity and insulin resistance through fat browning and alternative macrophage activation in mice fed a high-fat diet. BMJ Open Diabetes Res Care. 2019 10; 7(1):e000783 10.1136/bmjdrc-2019-000783 31749970 PMC6827766

[53] Bavdekar A, Yajnik C, Fall C, Bapat S, Pandit A, Deshpande V, et al Insulin resistance syndrome in 8-year-old Indian children: small at birth, big at 8 years, or both? Diabetes. 1999; 48:2422–2429 10.2337/diabetes.48.12.2422 10580432

[54] Surwit R, Kuhn C, Cochrane C, McCubbin J, Feinglos M. Diet-induced type II diabetes in C57BL/6J mice. Diabetes.1988; 37:1163–1167. 10.2337/diab.37.9.1163 3044882

[55] Pagliassotti MJ, Prach PA, Koppenhafer TA, Pan DA. Changes in insulin action, triglycerides, and lipid composition during sucrose feeding in rats. Am J Physiol. 1996; 271: R1319–R1326 10.1152/ajpregu.1996.271.5.R1319 8945970

[56] Beery AK, Zucker I. Sex bias in neuroscience and biomedical research. Neurosci Biobehav Rev. 2011;35(3):565–572. 10.1016/j.neubiorev.2010.07.002 20620164 PMC3008499

[57] Pessin J, Marts SA. Sex, gender, drugs, and the brain. Endocrinology. 2005; 146:1649 10.1210/en.2005-0198 15769898

[58] Sheard NF, Clark NG, Brand-Miller JC, Franz MJ, Pi-Sunyer FX, Mayer-Davis E, et al Dietary Carbohydrate (Amount and Type) in the Prevention and Management of Diabetes. A statement by the American Diabetes Association. Diabetes Care. 2004; 27(9): 2266–2271. 10.2337/diacare.27.9.2266 15333500

[59] Roncal-Jimenez CA, Lanaspa MA, Rivarda CJ, Nakagawa T, Sanchez-Lozada LG, Jalal D, et al Sucrose induces fatty liver and pancreatic inflammation in male breeder rats independent of excess energy intake. Metabolism. 2011; 60:1259–1260. 10.1016/j.metabol.2011.01.008 21489572 PMC3137694

[60] Klurfeld DM. Fructose: Sources, Metabolism, and Health. Encyclopedia of Food and Health, Reference Module in Food Science. 2016:125–129.

[61] Zhang F, Yuan W, Wei Y, Zhang D, Duan Y, Li B, et al The alterations of bile acids in rats with high-fat diet/streptozotocin-induced type 2 diabetes and their negative effects on glucose metabolism. Life Sci. 2019; 229:80–92. 10.1016/j.lfs.2019.05.031 31095947

[62] van den Berghe G, Bronfman M, Vanneste R, Hers HG. The mechanism of adenosine triphosphate depletion in the liver after a load of fructose. A kinetic study of liver adenylate deaminase. Biochem J. 1977; 162:601–609. 10.1042/bj1620601 869906 PMC1164643

[63] Price KL, Sautin YY, Long DA, Zhang L, Miyazaki H, Mu W, et al Human vascular smooth muscle cells express a urate transporter. J Am Soc Nephrol. 2006; 17:1791–1795. 10.1681/ASN.2006030264 16775029

[64] Wolk A, Furuheim M, Vessby B. Fatty acid composition of adipose tissue and serum lipids are valid biological markers of dairy fat intake in men. J Nutr. 2001; 131: 828–833. 10.1093/jn/131.3.828 11238766

[65] Antunes MM, Godoy G, de Almeida-Souza CB, da Rocha BA, da Silva-Santi LG, Masi LN, et al A high-carbohydrate diet induces greater inflammation than a high-fat diet in mouse skeletal muscle. Braz J Med Biol Res. 2020, 53(3), e9039 10.1590/1414-431X20199039 32077465 PMC7025447

[66] Institute of Medicine. Dietary Reference Intakes for Energy, Carbohydrate, Fiber, Fat, Fatty Acids, Cholesterol, Protein, and Amino Acids [Internet]. Washington, DC, National Academies Press, 2005 Available from: https://www.nap.edu/catalog/10490/dietaryreference-intakes-for-energy-carbohydrate-fiber-fatfatty-acids-cholesterol-protein-and-amino-acids. Accessed 4 December 2019.

[67] Evert AB, Dennison M, Gardner CD, Garvey GT, Lau KHK, MacLeod J, et al Nutrition Therapy for Adults with Diabetes or Prediabetes: A Consensus Report. Diabetes Care. 2019 5; 42(5): 731–754. 10.2337/dci19-0014 31000505 PMC7011201

[68] Salas-Salvado J, Bullo M, Estruch R, Ros E, Covas MI, Ibarolla-Jurado M, et al Prevention of diabetes with Mediterranean diets: a subgroup analysis of a randomized trial. Ann Intern Med. 2014;160:1–10. 10.7326/M13-1725 24573661

[69] Chiu THT, Pan W-H, Lin M-N, Lin C-L. Vegetarian diet, change in dietary patterns, and diabetes risk: a prospective study. Nutr Diabetes. 2018;8:12 10.1038/s41387-018-0022-4 29549240 PMC5856738

[70] van Zuuren EJ, Fedorowicz Z, Kuijpers T, Pijl H. Effects of low-carbohydrate- compared with low-fat-diet interventions on metabolic control in people with type 2 diabetes: a systematic review including GRADE assessments. Am J Clin Nutr. 2018;108:300–331. 10.1093/ajcn/nqy096 30007275

[71] Wheeler ML, Dunbar SA, Jaacks LM, Karmally W, Mayer-Davis EJ, Wylie-Rosett J, et al Macronutrients, food groups, and eating patterns in the management of diabetes: a systematic review of the literature, 2010. Diabetes Care. 2012;35:434–445. 10.2337/dc11-2216 22275443 PMC3263899

[72] Masharani U, Sherchan P, Schloetter M, Stratford S, Xiao A, Sebastian A, et al Metabolic and physiologic effects from consuming a hunter-gatherer (Paleolithic)-type diet in type 2 diabetes. Eur J Clin Nutr. 2015;69: 944–948. 10.1038/ejcn.2015.39 25828624

[73] Segain JP, De La Blatiere DR, Bourreille A. Butyrate inhibits inflammatory responses through NFK-B inhibition: implications for Crohn’s disease. Gut. 2000; 47(3):397–403. 10.1136/gut.47.3.397 10940278 PMC1728045

[74] Aziz AA, Kenney LS, Goulet B. Dietary starch type affects body weight and glycemic control in freely fed but not energy-restricted obese rats. J Nutr. 2009; 139(10):1881–1889. 10.3945/jn.109.110650 19692526

[75] Dehghan P, Pourghassem Gargari B, Jafarabadi MA. Oligofructose-enriched inulin improves some inflammatory markers and metabolic endotoxemia in women with type 2 diabetes mellitus: a randomized controlled clinical trial. Nutrition. 2014; 30(4):418–423. 10.1016/j.nut.2013.09.005 24332524

[76] Bendsen NT, Christensen R, Bartels EM, Astrup A. Consumption of industrial and ruminant trans fatty acids and risk of coronary heart disease: a systematic review andmeta-analysis of cohort studies. Eur J Clin Nutr 2011;65:773–783. 10.1038/ejcn.2011.34 21427742

[77] Landis SC, Amara SG, Asadullah K, Austin CP, Blumenstein R, Bradley EW, et al. A call for transparent reporting to optimize the predictive value of preclinical research. Nature. 2012; 490:187–191. 10.1038/nature11556 23060188 PMC3511845

