# Supplementary material for: Effects of the amount and type of carbohydrates used in type 2 diabetes diets in animal models: A systematic review
Source: PLoS One. 2020 Jun 12;15(6):e0233364. doi: 10.1371/journal.pone.0233364 (PMC7292416; doi:10.1371/journal.pone.0233364)
Supplement: S2 Table — (DOCX) [file pone.0233364.s002.docx]

**Supplementary material 2 (S2):** Table of experimental models.

**Table S2.** General characteristics of the experimental models used in all studies included in the systematic review.

| **Study** | **Species** | **Lineage** | **Sex** | **Weight**  **(g)** | **Age**  **(weeks)** | **Diet composition** | **Daily caloric intake (kcal)** | **Duration of the diet (weeks)** |
| --- | --- | --- | --- | --- | --- | --- | --- | --- |
| **High carbohydrate diet** |  |  |  |  |  |  |  |  |
| Bhathena *et al*., 1989 | *Rattus norvegicus* | SHR/N-cp/cp | Male | - | 5 | 54% sucrose  54% starch | - | 15 |
| Velasquez *et al*., 1995 | *Rattus norvegicus* | SHR/N-cp | Male | 385-605 | 6 | 54% CHO:  Sucrose or starch | - | 36 |
| Kazumi *et al*., 1997 | *Rattus norvegicus* | Wistar | Male | 431 | 18 | Chow and 10% glucose or fructose in water | 153 | 3 |
| Patel *et al*., 2009 | *Rattus norvegicus* | Wistar | Male | 320-350 | 8-9 | 61% Fructose  61% Cornstarch | 97 | 16 |

**Table S2 *(continuation)*.** General characteristics of the experimental models used in all studies included in the systematic review.

| **Study** | **Species** | **Lineage** | **Sex** | **Weight**  **(g)** | **Age**  **(weeks)** | **Diet composition** | **Daily caloric intake (kcal)** | **Duration of the diet** |
| --- | --- | --- | --- | --- | --- | --- | --- | --- |
| **High carbohydrate diet** |  |  |  |  |  |  |  |  |
| Bolsinger *et al*., 2013 | *Arvicanthis niloticus* | Nile | Male | 36-46 | 4 | HC: 70% CHO  MC: 40% CHO  LC: 10% CHO  HC+High Fibre: 70% CHO | 30-45 | 7 |
| Nojima *et al*., 2013 | *Mus musculus* | Nagoya-Shibata-Yasuda | Male | 15-20 | 4 | 47,8% CHO  High sucrose group: 30% sucrose  High fat: 50% fat | - | 12 |
| Parkman *et al*., 2016 | *Mus musculus* | TALLYHO | - | 22,8-34,3 | 3-4 | High sucrose low fat: 70,8% CHO  High sucrose high fat: 56,7% CHO | 9,6-13,92 | 28 |
| Arimura *et al*., 2017 | *Mus musculus* | C57BLKS | Male | 15-25 | 4 | Low protein: 71% CHO  High protein: 59% CHO | 7,1 | 2 |

**Table S2 *(continuation)*.** General characteristics of the experimental models used in all studies included in the systematic review.

| **Study** | **Species** | **Lineage** | **Sex** | **Weight**  **(g)** | **Age**  **(weeks)** | **Diet composition** | **Daily caloric intake (kcal)** | **Duration of the diet** |
| --- | --- | --- | --- | --- | --- | --- | --- | --- |
| **High carbohydrate diet** |  |  |  |  |  |  |  |  |
| Zhuo *et al*., 2018 | *Rattus norvegicus* | Wistar | Male | 180-220 | 8 | 61% CHO being 20% sucrose | - | 8 |
| Arimura *et al*., 2018 | *Mus musculus* | C57BLKS | Male | 15-25 | 5 | Low protein: 71% CHO, 12% Protein  High protein: 59% CHO, 24% Protein | 25,74-81,5 | 6 |
| Zhou *et al*., 2015 | *Rattus norvegicus* | Sprague-Dawley | Male | 180-200 | - | 80% CHO being 8% Resistant starch | - | 4 |
| Hedemann *et al*., 2017 | *Rattus norvegicus* | Zucker Diabetic Fatty | Male | - | 5 | 52,95% CHO:  Cornstarch, GLU, EMS or  resistant starch | - | 9 |

**Table S2 *(continuation)*.** General characteristics of the experimental models used in all studies included in the systematic review.

| **Study** | **Species** | **Lineage** | **Sex** | **Weight**  **(g)** | **Age**  **(weeks)** | **Diet composition** | **Daily caloric intake (kcal)** | **Duration of the diet** |
| --- | --- | --- | --- | --- | --- | --- | --- | --- |
| **Moderate carbohydrate diet** |  |  |  |  |  |  |  |  |
| Noonan & Banks, 2000 | *Mus musculus* | C57BL/6J | Male | 15 | 4 | 54% CHO, 5% fat  35% CHO (sucrose), 35% fat | 62,4-114,8 | 24 |
| Iwama *et al*., 2003 | *Rattus norvegicus* | Wistar | Male | 230 | - | 30% CHO (sucrose) | - | 4 |
| Shen *et al*., 2011 | *Rattus norvegicus* | GK | Female | 80-100 | 5 | 30% CHO (Resistant starch) | 57,68 | 10 |

**Table S2 *(continuation)*.** General characteristics of the experimental models used in all studies included in the systematic review.

| **Study** | **Species** | **Lineage** | **Sex** | **Weight**  **(g)** | **Age**  **(weeks)** | **Diet composition** | **Daily caloric intake (kcal)** | **Duration of the diet** |
| --- | --- | --- | --- | --- | --- | --- | --- | --- |
| **Low carbohydrate diet** |  |  |  |  |  |  |  |  |
| Pascoe *et al.*, 1992 | *Rattus norvegicus* | Wistar | Male | 343-359 | 8 | 20% CHO (High fat)  69% CHO (Starch) | 74 | 3 |
| Surwit *et al*., 1995 | *Mus musculus* | C57BL/6J | Male | 26,9-47,3 | 5 | 25% CHO (High fat):  HSHFD, LSHFD  73% CHO (Low fat):  LSLFD, HSLFD | 27-32 | 16 |
| Kaneko *et al*., 2000 | *Rattus norvegicus* | OLETF | Male | 470-800 | 10 | 80% CHO  60% CHO  40% CHO  20% CHO | - | 30 |
| Wang *et al*., 2003 | *Rattus norvegicus* | Wistar | Male | 400 | 9 | 60% CHO, 15% fat  10% CHO, 65% fat | 60 | 64 |
| Petro *et al*., 2004 | *Mus musculus* | C57B1/6J | Male | 15 | 4 | 73% CHO, 11% fat  26% CHO, 58% fat | 7,1-8,7 | 11 |
| Asghar *et al*., 2006 | *Mus musculus* | MKR | Male | 10-15 | 5 | 12% CHO (sucrose), 58% fat | 3,9-6,6 | 18 |
| Marsh *et al*., 2009 | *Rattus norvegicus* | Sprague-Dawley | Male | 270-645 | 6 | Control: 69% CHO  High fat: 21% CHO  Western diet: 45% CHO | - | 12 |
| Sun *et al*., 2018 | *Rattus norvegicus* | Sprague-Dawley | Male | 180-200 | 8 | Resistant starch: 10%, 15% and 20% | - | 4 |

g = grams; - = missing info; CHO = carbohydrate; HC = high carbohydrate; MC = moderate carbohydrate; LC = low carbohydrate; GLU = glucidex; EMS = enzimatically modified starch; HSHFD = high sucrose high fat diet; LSHFD = low sucrose high fat diet; LSLFD = low sucrose low fat diet; HSLFD = high sucrose low fat diet.
